# A Pilot Study Investigating a Novel Non-Linear Measure of Eyes Open versus Eyes Closed EEG Synchronization in People with Alzheimer’s Disease and Healthy Controls

**DOI:** 10.3390/brainsci8070134

**Published:** 2018-07-17

**Authors:** Daniel J. Blackburn, Yifan Zhao, Matteo De Marco, Simon M. Bell, Fei He, Hua-Liang Wei, Sarah Lawrence, Zoe C. Unwin, Michelle Blyth, Jenna Angel, Kathleen Baster, Thomas F. D. Farrow, Iain D. Wilkinson, Stephen A. Billings, Annalena Venneri, Ptolemaios G. Sarrigiannis

**Affiliations:** 1 Department of Neuroscience, University of Sheffield, Sheffield S10 2HQ, UK; m.demarco@sheffield.ac.uk (M.D.M.); s.m.bell@sheffield.ac.uk (S.M.B.); t.f.farrow@sheffield.ac.uk (T.F.D.F.); a.venneri@sheffield.ac.uk (A.V.); 2 Department of Clinical Neurophysiology, Sheffield Teaching Hospitals NHS Foundation Trust, Sheffield S10 2JF, UK; yifan.zhao@cranfield.ac.uk; 3 School of Aerospace, Transport and Manufacturing, Cranfield University, Cranfield MK43, UK; fei.he@imperial.ac.uk; 4 School of Mathematics & Statistics, University of Sheffield, Sheffield S1 2TN, UK; w.hualiang@sheffield.ac.uk (H.-L.W.); s.billings@sheffield.ac.uk (S.A.B.); 5 Academic Unit of Radiology, University of Sheffield, Sheffield S1 2TN, UK; Sarah.Lawrence@sth.nhs.uk (S.L.); Zoe.Unwin@sth.nhs.uk (Z.C.U.); Michelle.Blythe@sth.nhs.uk (M.B.); Jenna.Angel@sth.nhs.uk (J.A.); p.sarrigiannis@sheffield.ac.uk (P.G.S.); 6 Department of Life Sciences, Faculty of Natural Sciences, Imperial College London, London SW7, UK; k.baster@sheffield.ac.uk; 7 Department of Automatic Control & Systems Engineering, University of Sheffield, Sheffield S10 2TN, UK; i.d.wilkinson@sheffield.ac.uk

**Keywords:** Alzheimer’s disease, electroencephalography, clinical marker, ROC curve, nonlinear dynamics

## Abstract

Background: The incidence of Alzheimer disease (AD) is increasing with the ageing population. The development of low cost non-invasive diagnostic aids for AD is a research priority. This pilot study investigated whether an approach based on a novel dynamic quantitative parametric EEG method could detect abnormalities in people with AD. Methods: 20 patients with probable AD, 20 matched healthy controls (HC) and 4 patients with probable fronto temporal dementia (FTD) were included. All had detailed neuropsychology along with structural, resting state fMRI and EEG. EEG data were analyzed using the Error Reduction Ratio-causality (ERR-causality) test that can capture both linear and nonlinear interactions between different EEG recording areas. The 95% confidence intervals of EEG levels of bi-centroparietal synchronization were estimated for eyes open (EO) and eyes closed (EC) states. Results: In the EC state, AD patients and HC had very similar levels of bi-centro parietal synchronization; but in the EO resting state, patients with AD had significantly higher levels of synchronization (AD = 0.44; interquartile range (IQR) 0.41 vs. HC = 0.15; IQR 0.17, *p* < 0.0001). The EO/EC synchronization ratio, a measure of the dynamic changes between the two states, also showed significant differences between these two groups (AD ratio 0.78 versus HC ratio 0.37 *p* < 0.0001). EO synchronization was also significantly different between AD and FTD (FTD = 0.075; IQR 0.03, *p* < 0.0001). However, the EO/EC ratio was not informative in the FTD group due to very low levels of synchronization in both states (EO and EC). Conclusion: In this pilot work, resting state quantitative EEG shows significant differences between healthy controls and patients with AD. This approach has the potential to develop into a useful non-invasive and economical diagnostic aid in AD.

## 1. Introduction

There is an urgent need for widely available low-cost diagnostic aids to detect cognitive impairment and early signs of Alzheimer’s disease (AD) [1]. The electroencephalogram (EEG) has been used clinically for many decades as a tool to help diagnose AD and other types of dementia. EEGs are economical, non-invasive and have no-contraindications. EEG studies have consistently shown abnormalities in AD patients; slowing of the posterior dominant rhythms and an increase in the slow wave activity is the most common feature on visual inspection of EEGs of patients with AD [2]. Spectral EEG analyses provide the means for a more objective estimation of the frequencies involved and for determining, to an extent, the spatial distribution of the abnormalities. Early changes in AD are an increase in theta and a decrease in beta activity [2]; these EEG abnormalities are also associated with cognitive deficits [2,3].

Advances in quantitative analysis from EEG recording (QEEG) have made it possible to detect changes in synchronization between different brain regions, reflecting underlying network dysfunction resulting from neuronal or synaptic death and loss of cholinergic inputs in this disease [2]. The characterization of EEG network alterations, therefore, holds promise to detect changes in people with AD [2,4,5]. QEEG studies report decreases in synchronization estimated with measures of coherence, between both contiguous and distant EEG channels in people with AD, suggesting functional disconnection among cortical regions [2,6,7,8]. Stam et al. [9] used a technique called synchronization likelihood, documenting decreased levels [10,11] as well as diminished fluctuations in synchronization [12]. This reduction especially affected alpha and beta bands, although gamma band decrease has also been reported [13]. The decrease in synchronization between brain areas may not be due to loss of cortical neurons, as not all frequencies are equally affected [2,10], and increased synchronization can also be found for slow rhythms [10]. Impairments in brain network function, as detected using QEEG (and other techniques), may be one of the earliest features of AD [14]; therefore, it can be a useful diagnostic aid as there is a need to detect AD as early as possible if clinicians are to intervene and test new treatments.

Normal cognitive function requires the integrity of functional network dynamics [4,15,16], and the study of oscillatory synchronization between different brain areas can provide information on functional network activity [4]. The study of brain synchronization utilizes a number of different techniques, such as measures of coherence (e.g., [17]). QEEG analysis of local and global networks (i.e., hubs and modules—used to define aspects of healthy brain functioning [5]) has detected disruption of brain networks in people with AD [5,11,18,19,20]. Networks that connect the parietal lobes appear to be particularly affected [21,22]. Hata et al. used 40 s epochs of EEG, recorded from people with AD, and found decreased lagged phase synchronization between the right dorsolateral prefrontal cortex and the right posterior-inferior parietal lobule within the theta band [22].

QEEG can also measure complexity of signal as well as brain synchronization or connectivity. Non-linearity is a necessary condition of the highly-complex nature of brain function. QEEG can be quantified with several nonlinear methodologies that may be advantageous in detecting brain network dysfunction. Abasolo et al. [23] used the SampEn nonlinear method and demonstrated significantly lower levels of complexity in the occipital and parietal lobes of patients with AD. Electrode O1 had the highest sensitivity (90.9%; ROC score = 89.3%) and P3 the highest specificity (90.9%; ROC score = 85.1%), producing a combined accuracy of 81.8% [23,24]. Abasolo also used another nonlinear methodology, the Lempel-Ziv complexity that further demonstrated significantly-lower complexity scores in AD patients [23]. The authors inferred that people with AD have more regular and less complex activity in posterior (parietal and occipital) brain regions. Their approach is based on single recording positions and does not provide information on the underlying functional connectivity between areas. Other authors have also demonstrated reduced brain complexity as measured using nonlinear methods in people with AD [2,25,26,27].

Despite evidence of altered brain synchronization and network dysfunction in people with AD, it is not clear which EEG states are most useful to sample: epochs of Eyes-Open (EO), Eyes-Closed (EC), or mixed EO-EC. Moreover, this procedural detail is often not reported in published studies. The evidence that patients with AD show loss of brain responsiveness to environmental stimuli [2,28] suggests that dynamic changes between EO and EC might represent an ideal paradigm to investigate the effect of AD pathophysiology. For example, Miraglia et al. [29] used graph theory analysis of EO versus EC QEEG to reveal changes of EEG reactivity.

We have developed a novel QEEG parametric data analysis technique—the Error Reduction Ratio-causality (ERR-causality) test [30]—that can measure levels of synchronization with high temporal resolution. The ERR-causality test makes no assumptions about the type of EEG interactions and is able to detect both linear and nonlinear synchronization. This analytic technique has been compared to other QEEG measures from sleep recordings and in patients with epilepsy ([30] and Appendix A), with favourable results.

In this pilot study, the ERR-causality method was applied to EEG recordings acquired in a cohort of patients with Alzheimer’s disease in the early dementia phase and healthy controls (HC) in order to measure linear and non-linear synchronization between the two hemispheres. We decided to select parietal lobe regions and focused on bi-hemispheric synchronization because there is a large body of available supporting evidence that reports early dysfunction or pathology in these areas in AD. Evidence includes findings from studies of brain metabolism [31], task fMRI activation [32], resting state fMRI [33,34] and QEEG studies of brain connectivity [5,11,18,19,20,21,22,23,24,35]. Furthermore, parietal areas are relatively spared by artifacts triggered by eye-blinking and/or temporal muscle contraction. As patients with AD show loss of brain responsiveness to environmental stimuli [2,28], both EC and EO states were acquired and analyzed. We also included a small group of patients with fronto temporal dementia (FTD) to rule out non specific effects of neurodegeneration. We hypothesized that estimates of EEG synchronization with the ERR test between the homologous centroparietal areas during EO and EC states would allow differentiation between patients with AD and healthy controls. We demonstrate in this pilot study that this novel approach reveals alterations in brain bi-hemispheric synchronization, and has the potential to become a useful diagnostic aid in people with cognitive impairment related to AD.

## 2. Materials and Methods

### 2.1. Study Design

All EEG data were acquired and analyzed prospectively. A task-free EEG that requires minimal cooperation was chosen as AD patients may have difficulty engaging with and following cognitive tasks. The parametric ERR-causality technique is a very dynamic method (resolution of 0.5 ms at a sampling rate of 2 KHz) that works very well on small, few-second epochs of EEG data without any assumptions regarding linear or nonlinear dynamic relationships between two time series. The data were not segmented into frequency bins because in nonlinear dynamic systems that can exhibit harmonics and inter-modulation effects. It is likely that exciting frequencies can be transferred by the nonlinear effects to other frequency locations and can, therefore, move energy between bins, that would consequently corrupt any subsequent analysis. In this study, synchronization estimates were produced between homologous centroparietal (and frontal for comparison) EEG areas. Other EEG electrode combinations were not analyzed.

### 2.2. Sample

All participants were recruited from Sheffield Teaching Hospitals NHS Foundation Trust memory clinics, and HC were enrolled through opportunistic sampling and word of mouth. The Sheffield Teaching Hospital memory clinic is a young-onset memory clinic, seeing people predominantly aged under 65. The study recruited participants from September 2014 to March 2017. The patient sample included 20 participants with a clinical diagnosis of AD and four with a clinical diagnosis of FTD. The participants with AD had been diagnosed 1 month to 2 years prior to this study and were in the mild to moderate stages of disease, with a mean Mini Mental State Examination (MMSE) score of 20.10 (standard deviation 4.00). The diagnosis of AD was based on the NINCDS-ADRDA criteria [36]; participants with FTD were diagnosed according to the Rascovsky criteria [37]. Diagnosis was reached based on a consensus of multidisciplinary evidence, taking into account clinical history, neurological examination, neuropsychological scores, and neuro-radiological findings.

Nine out of the 20 patients with AD were scanned with technetium-99-hexamethylpropylene amine oxime (HMPAO) SPECT imaging that revealed parietal and temporal lobe hypoperfusion consistent with AD. All participants with AD were on standard treatment—17 were taking acetylcholinesterase inhibitor medication (AChEI; 13 taking Donepezil, one Galantamine and one taking Rivastigmine) and three were taking Memantine; two received memantine because they did not tolerate initial treatment with an AChEI and one for cardiac contraindication that precluded using an AChEI). All EEGs were performed while patients were taking medication at a stable dose. Six patients were also on anti-depressants, with one additionally receiving an anti-epileptic medication for myoclonus (levetiracetam). One further participant (not on anti-depressants) received anti-epileptic medication for seizures (lamotrigine). No participants were taking benzodiazepines. None of the patients with FTD were on any psychoactive medication.

Data were also collected from a sample of 20 age and gender matched healthy controls. Everyone underwent an extensive battery of neuropsychology testing, and structural and functional MRI imaging (see below).

### 2.3. Magnetic Resonance Imaging Acquisition

All patients were scanned with high-resolution structural MRI to rule out major etiologies that could otherwise account for their clinical symptoms. No patient had significant small vessel ischemic disease, as supported by a fluid-attenuated inversion recovery (FLAIR) MRI scan acquisition.

Each participant consented to undergo a 3 T (Philips Ingenia) MRI scanning protocol, which included structural images and resting-state functional scans. Additional diffusion, T2 weighted and FLAIR acquisitions served to verify/corroborate the absence of exclusion criteria. MRI assessments were obtained up to an average of 79 days prior to study inclusion. Structural and functional neuroimaging served for the sole purpose of characterizing brain structure and brain function in the group of patients, to ensure that they were typical of the clinical population object of this investigation.

Anatomical T1-weighted volumes were acquired tri-dimensionally with the following parameters—voxel dimension: 0.94 × 0.94 × 1.00 mm, field of view: 256 mm, matrix size: 256 × 256 × 124, repetition time = 8.2 s, echo delay time 3.8 ms, flip angle: 8°. Following a series of dummy scans set up to allow electromagnetic equilibrium, a T2*-weighted scan was recorded to measure cerebral hemodynamics at rest. This scan included 120 volumes, each of which consisted of 35 axial slices, contiguously acquired in ascending order. Participants were invited to close their eyes for the whole duration of the scan. The following specifics were set up—repetition time = 2.6 s, total acquisition time: 4 m and 30 s, echo delay time = 35 ms, flip angle: 90°, voxel dimensions: 2.4 × 2.4 × 4.0 mm, field of view: 230 × 230 × 140 mm.

### 2.4. Magnetic Resonance Imaging Analysis

All MRI images were preprocessed and modelled with SPM12b software (Wellcome Centre for Human Neuroimaging, London, UK), implemented in a Matlab environment (vR2011b, The Mathworks, Natick, MA, USA).

Following voxel-brain-morphometry procedures [38], T1-weighted images were initially segmented to separate gray matter (GM) and white matter (WM) from non-neural tissue classes. GM maps were then registered to the Montreal Neurological Institute space, and were smoothed with an 8 mm^3^ full-width at half maximum Gaussian kernel. Furthermore, unprocessed images were elaborated with the fully-automatized “STEPS” procedure (available online at http://cmictig.cs.ucl.ac.uk/niftyweb/), which, thanks to the optimized use of multiple templates, enables an accurate segmentation of the left and right hippocampus [39]. All native-space output sub-maps were processed with the “get_totals” script (www.cs.ucl.ac.uk/staff/g.ridgway/vbm/get_totals.m) for the calculation of absolute and fractional volumes of the hippocampus and global maps of GM and WM. Absolute volumes were expressed in milliliters. Fractional volumes were obtained by dividing each tissue-class volume by the total intracranial volume (TIV), which was computed, in turn, by adding up global volumes of GM, WM, and cerebrospinal fluid.

A standard pipeline was chosen to preprocess resting-state functional scans. Volumes were initially slice-timed and realigned. The graphical output of realignment was visually inspected to ascertain that no participant showed excessive in-scanner motion. Realigned runs were then spatially normalized using the default echoplanar SPM template, and voxel size was rendered isotropic at 2.0 × 2.0 × 2.0 mm. After normalization, volumes were band-pass filtered (0.01 Hz–0.1 Hz) to eliminate frequencies not believed to be of neurogenic origin. For this purpose, the REST toolbox (www.restfmri.net) was used [40]. Images were finally smoothed with a 6 mm^3^ full-width at half maximum Gaussian kernel.

For a characterization of default-mode network (DMN) hemodynamic circuitry, brain networks were computed with a group-level independent component analysis [41], using the GIFT toolbox (v1.3i, mialab.mrn.org/software/gift). Independent component analysis separates latent sources of variability based on the matrix of correlations among the measured variables (the time-course recorded in each voxel). The Infomax algorithm was chosen in order to minimize mutual information of components, and the number of components to estimate was set at 20. The results were visually inspected and, upon agreement between two independent raters (MDM and AV), the DMN was selected among the 20 components based on its map, which normally includes the posterior cingulate, the medio-prefrontal region, the inferior parietal lobule, the lateral temporal cortex and the hippocampal formation [42].

### 2.5. EEG Recordings

EEG recordings were acquired with a modified 10/10 overlapping a 10/20 international system of electrode placement method. All recordings were made with an XLTEK 128-channel headbox (Optima Medical LTD, Surrey, UK) at a sampling rate of 2000 Hz (analogue bandwidth 0.15–680 Hz) with scalp Ag/AgCL electrodes. A linked earlobe reference was used for all participants (jump cables were used to combine both electrodes into one input; care was taken for impedance to be equal on both sides). A 30-min resting state EEG recording was obtained, including alternating 5-min EO and EC epochs (during which the participants were encouraged not to think about anything specific). Thirty minutes is longer that the analysis requires, but allows representative epochs to be selected. If participants showed signs of drowsiness, they were prompted.

An experienced neurophysiologist reviewed all EEGs on the XLTEK review station with time-locked video recordings (Optima Medical LTD) with a bipolar montage that included the following channels: Fp2-F8, Fp1-F7, F8-F4, F7-F3, F4-C4, F3-C3, F4-FZ, FZ-CZ, F3-FZ, T4-C4, T3-C3, C4-CZ, C3-CZ, CZ-PZ, C4-P4, C3-P3, T4-T6, T3-T5, P4-PZ, P3-PZ, T6-O2, T5-O1, P4-O2, P3-O1 and O2-O1. Two 12-s artefact free epochs (one EO and one EC) were selected. For consistency, the first 12-s epoch for each patient for EO and EC was used for further analysis. Data were exported in Spike 2 (version 8) software and all the aforementioned EEG bipolar derivations were produced and then processed with a time constant of 0.08 (high pass filter at 2 Hz), to attenuate any remaining low frequency artifacts like those generated by eye blinking and slow movements.

The ERR-causality synchronization estimates were produced using an in-house software developed by a complex-signal analysis engineering team. Briefly, the ERR is an extension of the Granger causality test and incorporates a model to characterize the causal interaction over time between two signals, denoted by X and Y. At a specific time, the following possibilities can occur: signal X causes Y, Y causes X, X and Y are coincident, or there is no interaction, or bidirectional interactions occur between them. Both X and Y may have been caused by an input u, but here u is unknown and not measurable. Steps involve: construct a candidate term set which is typically constructed by past information of Y, for example y(t − 1),y(t − 12),…and past information of X, for example x(t − 11), x(t − 12), ... with a specific model order d. Apply the adaptive-forward-OLS algorithm which has been derived from the orthogonal least squares (OLS) algorithm [43] and compute ERR and the penalized error-to-signal ratio PESR) [44] value for each candidate term. ERR indicates how much of the variance change in the system response is caused by the considered term, expressed as percentage. If the significant terms selected by this algorithm based on values of ERR include any term from the past information of X, this indicates that signal X leads Y during the considered time duration (t − 1h/2, t + h/2), where h denotes the sampling window size. This was selected at 1000 data points (corresponding to 0.5 s) for this study. The ERR-causality from X to Y at time t, expressed as F_x_ > y(t) is then defined as 1. If no component from the past information of X is included in the selected significant terms, this indicates that X has no interaction with Y during [t − h/2, t + h/2] and F_x_ > y(t) is defined to be 0. The strength of F_x_ > y(t) can be estimated using the summed ERR values of all the selected terms from past information of X, the maximum strength being 1. The time shift of X > Y is defined as the time lag of X in the first term ranked by the values of ERR. For a complex system, the causality is often time varying and the interactions are often dynamic and nonlinear. Linear and nonlinear interactions can be separated and analyzed independently with this method, which is relevant as the nonlinear dimension appears to be important in the pathophysiology of AD and cannot be assessed by conventional QEEG methods [2,25]. Further details of the ERR methodology are included in Appendix A.

**Figure 1:**
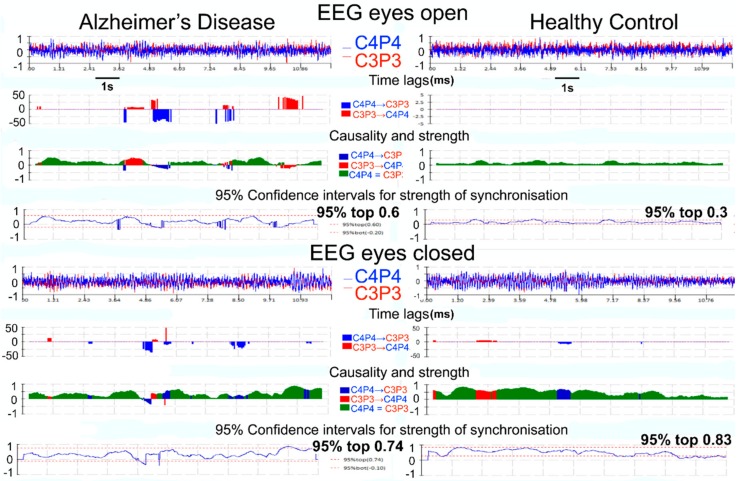
This figure illustrates the results of our novel QEEG method, the EER-causality test. The figure includes screen shots taken from the software results screen. The data included are from one participant with AD on the left and a HC on the right. The top half of the panel is from the eyes open (EO) state and lower panel from eyes closed (EC) state. Each EC and EO resting state contains 12 s EEG epochs. For each EO and EC state, AD (**left**) and HC (**right**), row one shows the normalized EEG data from the homologous centroparietal areas, row two the time lag estimates (not used in our current work), row three the strength of synchronization (causality: degree of association between the centroparietal EEG time series), and row four the 95% confidence intervals for the strength of synchronization. The color-coding indicates the direction of synchronization; blue = right drives left, red = left drives right, green = isochronous zero-lag synchronization. Please note that in the EO state, there are dynamic fluctuations in the level of synchronization with peaks of causality exceeding 0.5 in the AD participant, not seen in the HC example. These fluctuating bursts of high-level synchronization are captured with our dynamic methodology and they are best expressed in the 95% confidence intervals of the strength of synchronization.

One EO and one EC 12-s epochs were selected for each subject to produce the 95% confidence intervals for EO and EC synchronization levels. The 95% confidence interval is used to measure synchronization variability. The 95% confidence interval is represented by µ ± 1.96σ, where µ and σ denote the mean and standard deviation of the samples, respectively. This was carried out on a 6-s period internal to the epoch, in order to enable visual assessment of the 95% confidence interval for the remaining 6 s for each case, both for EO and EC states (Figure 1). 

We analyzed bifrontal synchronization using the F8-F4 and F7-F3 derivations as a control area, as this region is less commonly affected in AD. The ratios of EO versus EC synchronization were calculated for all participants with the ERR-causality test. Although the investigators in charge of carrying out the ERR-causality method were aware of the clinical diagnosis during data analysis, selection bias was avoided by consistently choosing the first, in chronological order, EO and EC epoch from each EEG recording.

### 2.6. Neuropsychological Assessment

The cognitive profile of AD patients and healthy controls was determined using an extensive battery of neuropsychological tests, specifically devised to be sensitive to cognitive difficulties typically triggered by AD [45]. This included the Mini Mental State Examination [46], tests of short and long-term memory (verbal and non-verbal) [47] tests of abstract reasoning [47,48] tests of attention and executive function [49], language comprehension, naming by confrontation, category and letter fluency [50] (a specific focus on the cognitive domains most relevant for the purpose of this investigation is given below in Table 1).

### 2.7. Statistical Methods

Summary statistics were calculated for each of the outcomes, split by group. Demographic data, global indices of absolute and fractional brain anatomy, and neuropsychology results were tested for selection bias (Wilcoxon Mann-Whitney test). Moreover, to ensure that AD patients did not move away from well-established patterns of disease effects, comparisons were run to model between-group differences in regional maps of GM and in maps of DMN connectivity (two-sample *t* tests). A cluster-level *p* value (corrected for Family-Wise Error) < 0.05 was set as threshold of significance. The nonlinear transform available at “http://imaging.mrc-cbu.cam.ac.uk/downloads/MNI2tal/mni2tal-m” was used to transpose Montreal National Institute peak coordinates into the Talairach Space. The Talairach Daemon Client [51], finally, served for interpretational purposes.

The QEEG results were tested for differences between the HC, AD and FTD groups using the Wilcoxon Mann-Whitney test for two independent samples. ROC curves were constructed using data from AD and HC groups, showing sensitivity and specificity for our proposed diagnostic tool.

This study was approved by the Yorkshire and The Humber (Leeds West) Research Ethics Committee (reference number 14/YH/1070). All participants gave their informed written consent.

## 3. Results

The demographic data, MRI and neuropsychology results are summarized in Table 1. This shows that HC and AD were both age and gender matched, but the HC did have a higher level of education. In support of a diagnosis of AD, all participants, including all HC, had detailed neuropsychology (Table 1), with AD subjects showing profiles consistent with AD and normal profiles in healthy controls.

### 3.1. MRI Results

**Figure 2:**
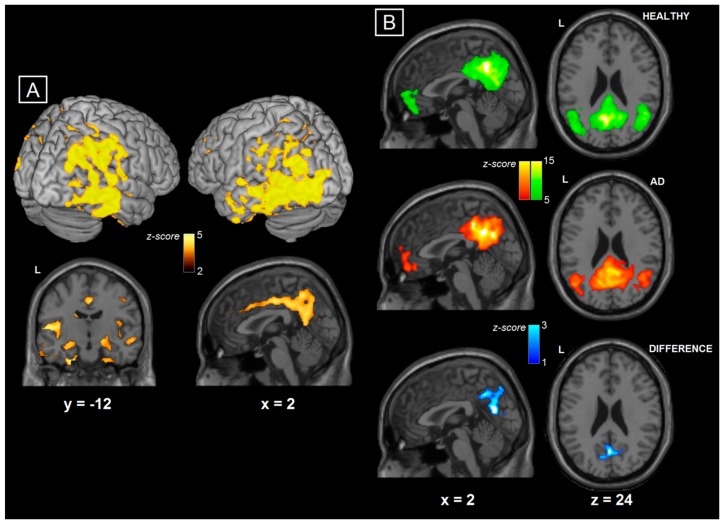
(**A**) Structural MRI, group comparison between AD and HC; Structural MRI comparison between the two diagnostic groups showing the regions where HC had larger grey-matter volumes than AD patients. Results show clusters surviving a Family-Wise-Error corrected cluster-level *p* < 0.05. Montreal Neurological Institute coordinates are indicated at the bottom of each slice. (**B**) Functional MRI, group comparison between AD and HC. fMRI comparison between AD and HC groups showing the regions where HC had more DMN fMRI connectivity than AD patients. Differences were visible in a cluster surviving a Family-Wise Error corrected cluster-level *p* < 0.05 extending to the posterior cingulate gyrus and precuneus (peaks in Brodmann Areas 7, 29 and 31). Montreal Neurological Institute coordinates are indicated in the figure. This finding is consistent with previous publications and shows loss of connectivity in the posterior part of the DMN.

Structural and functional MRI analyses revealed patterns of atrophy and dysfunction as expected in AD, suggesting that the sample selected for this study was typical of the AD population of mild to moderate severity. In detail, significantly smaller mean gray matter volume was detected in the group of AD patients (Table 1). Voxel-based morphometry analysis showed decreased gray-matter volumes in patients in a series of neocortical regions, including the parietal, prefrontal, and temporal lobes. Moreover, bilateral volumetric decrease was found in the hippocampus and overall in the mediotemporal complex (Figure 2A). Analysis of resting state fMRI showed that patients with AD had significantly reduced DMN connectivity in a cluster located in the posterior cingulate/precuneus, when compared to controls (Figure 2B)

### 3.2. QEEG Results

**Figure 3:**
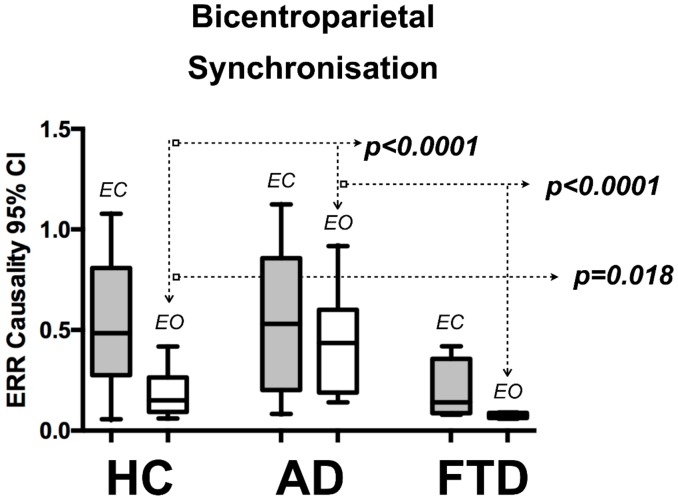
QEEG bi-centroparietal synchronization, group comparison between AD, HC & FTD. This graph depicts the median with 95% CI bi-centroparietal (C3–P3 to C4–P4) synchronization for Eyes Closed (EC) and Eyes Open (EO) states from HC-Healthy Controls (*n* = 20) and AD-Alzheimer’s disease (*n* = 20) & FTD–frontotemporal dementia (*n* = 4). The non-parametric Mann Whitney test of group differences was used.

The QEEG results were not normally distributed. Hence, non-parametric tests were used to compare the two groups. QEEG measurements of EC bi-centroparietal synchronization showed no significant difference between participants with AD and HC. However, patients with AD had higher levels of median synchronization during EO (AD 0.44 vs. HC 0.15, *p* < 0.0001), Table 2 and Figure 3.

**Figure 4:**
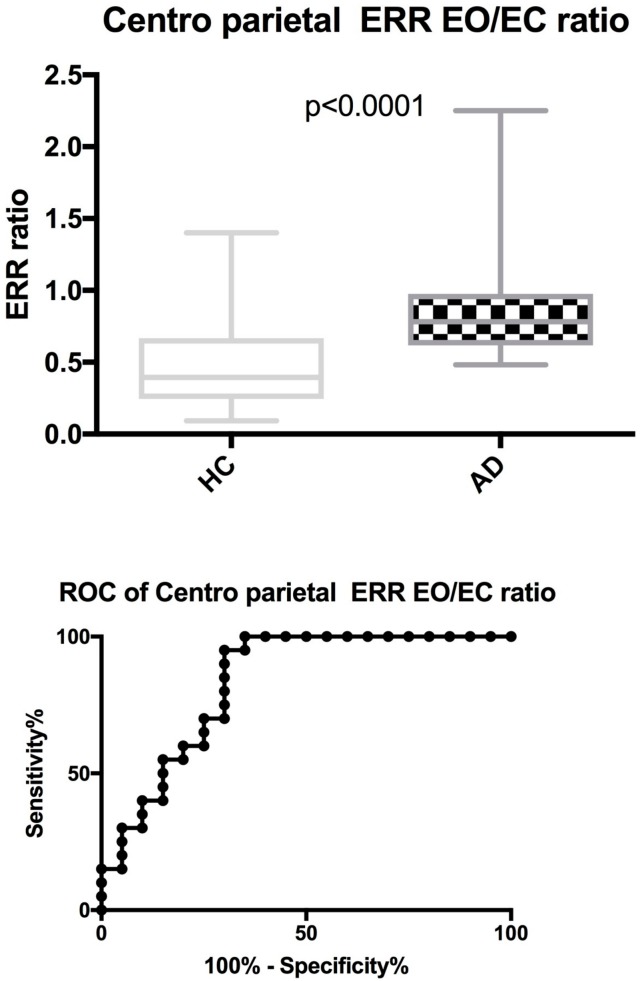
QEEG EO/EC bi-centroparietal synchronization ratio, group comparison between AD and HC (including ROC). This figure depicts the EO/EC bi-centroparietal synchronization ratios for HC (Healthy Controls) and AD (Alzheimer disease). The ROC curve represents a cut-off point at 0.51 for all participants producing a sensitivity of 95% and specificity of 75%.

Interestingly, the ratio of EO versus EC synchronization, a measure of dynamic changes between two states, shows a significant difference between AD and HC (AD 0.78 vs. HC 0.37, *p* < 0.0001, Table 2 and Figure 4). An EO/EC bi-centroparietal ratio of 0.51 produces sensitivity and specificity of 95% and 75%, respectively. Even after correcting for education levels, these results retained their significance. The age range of our HC cohort was between 48–86 years, with 10 participants above the age of 70.

**Figure 5:**
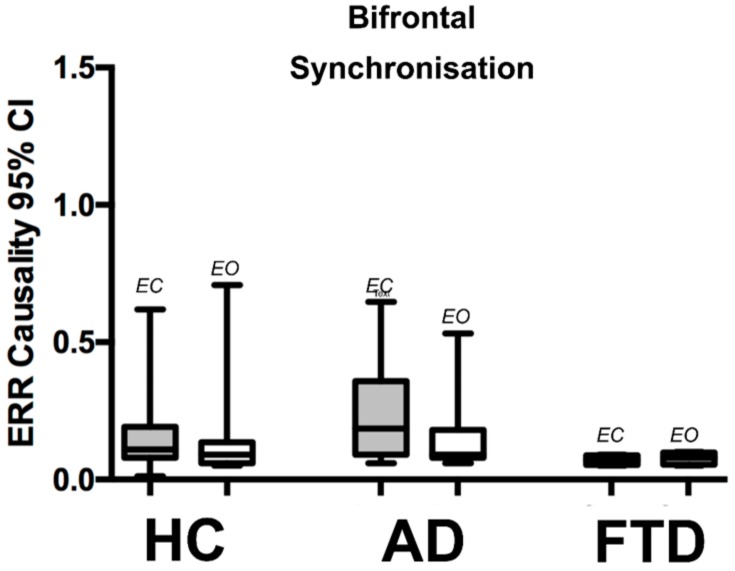
QEEG bi-frontal synchronization, group comparison between AD & HC. This graph depicts the median with 95% CI bi-frontal (F3–F7 to F4–F8) synchronization for Eyes Closed (EC) and Eyes Open (EO) states from three groups; HC-Healthy Controls (*n* = 20), AD—Alzheimer’s disease (*n* = 20), FTD—Fronto-Temporal Dementia (*n* = 4). The graph shows no significant differences in the degree of synchronization measurements for each group and, additionally, for each state (i.e., EO & EC), there is no difference within each group.

EO versus EC synchronization was also tested for comparison between homologous frontal lobes as these are relatively spared in AD. We estimated synchronization between F8–F4 & F7–F3. This showed no significant difference in either state (EO or EC) between AD and HC. There was also no dynamic change observed between the EO and EC states for HC and AD (see Table 2 and Figure 5).

The patterns of bi-centroparietal synchronization seen in patients with FTD differ from those seen in patients with AD. Specifically, participants with FTD have much lower EO bi-centroparietal synchronization when compared to the AD groups (0.08 vs. 0.44, *p* < 0.001) (see Table 2). The FTD cohort also had significantly lower levels of EO bi-centroparietal synchronization compared to healthy controls (0.08 vs. 0.15 *p* = 0.018 and EC state 0.14 (IQR 0.27) vs. 0.484 (IQR 0.53), *p* = 0.018, see Table 2). The EO/EC bi-centroparietal synchronization ratio appears to be not informative in the FTD group because minimal levels of synchronization were measured from both EO and EC states.

**Figure 6:**
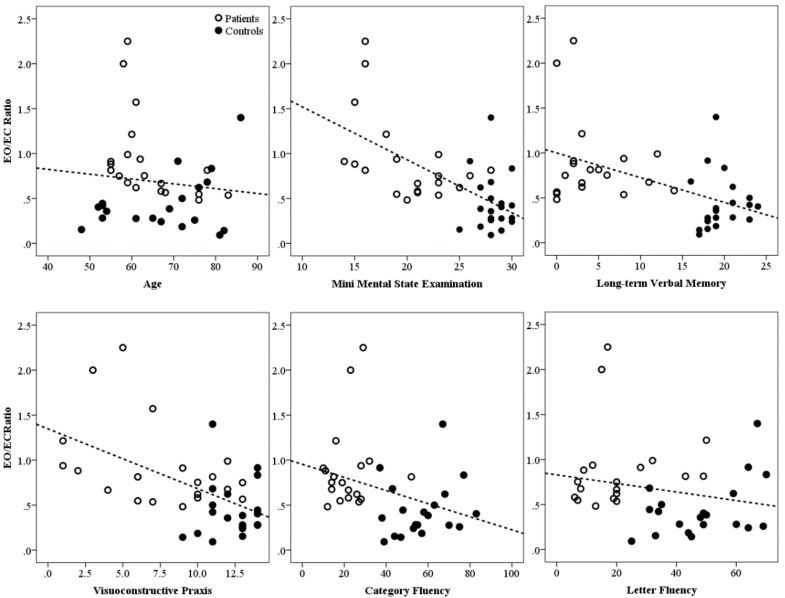
Electroclinical (neuropsychology) correlations. Relationship between EO/EC bi-centroparietal synchronization ratio and a series of variables, including age, general cognitive level, verbal episodic memory, visuoconstructive abilities, and verbal fluency. Although these analyses were carried out using non-parametric rank-based correlations (see Results section), a linear model was fitted for the sole purpose of visualization. The r correlations (perfectly mirroring nonparametric statistics) are as follows—Age: *r* = −0.116, *p* = 0.475; MMSE: *r* = −0.629, *p* < 0.001; long-term verbal memory: *r* = −0.508, *p* = 0.001; visuoconstructive praxis: *r* = −0.537, *p* < 0.001; category fluency: *r* = −0.338, *p* = 0.035; letter fluency: *r* = −0.205, *p* = 0.211.

Finally, electro-clinical correlations were explored by assessing the relationship between EO/EC bi-centroparietal synchronization ratio for each participant against a series of factors, including age, MMSE, and the cognitive tests most frequently used in clinical settings to aid a diagnosis of AD (Figure 6 and Table 2). For this purpose, a non-parametric coefficient of correlation (Spearman’s rank correlation coefficient, rho) was chosen and a *p* < 0.01 was used. Age was not correlated with EO/EC ratio (*rho* = −0.074, *p* > 0.01). The association between EO/EC bi-centroparietal synchronization ratio and the MMSE score was highly significant (*rho* = −0.606, *p* < 0.001). Significant correlations were also found between the EO/EC bi-centroparietal synchronization ratio and test performance in various cognitive domains (see Figure 6): memory (Prose Memory Test-Immediate Recall: *rho* = −0.537, *p* < 0.001; Prose Memory Test-Delayed Recall: *rho* = −0.489, *p* = 0.002), semantic processing (Palm Trees Test: *rho* = −0.616, *p* = 0.004; Category Fluency Test: *rho* = −0.767435, *p* < = 0.001, visuospatial abilities (Visuoconstructional Praxis Test: *rho* = −0.704448, *p* < = 0.001004), and language comprehension (Token Test: *rho* = −0.875, *p* < 0.001). However, the bi-centroparietal synchronization ratio did not correlate with executive functioning (Stroop Test-Time Interference: *rho* = 0.134, *p* = 0.429; Letter Fluency Test: *rho* = −0.229, *p* = 0.162) that predominantly relies on frontal lobe function. This shows concordance between location of cognitive function testing and our electrophysiological measure.

## 4. Discussion

The results from this small pilot study are encouraging and show a significant difference in EO bi-centroparietal synchronization between people with mild to moderate Alzheimer’s disease and healthy controls. The relative young age of our AD cohort (mean age of 63.95) reflects the referral pattern to a young onset memory service (commonly patients younger than 65 are referred to our clinic). To safeguard against any spurious demographic effect, the healthy control and AD sample were age and gender matched in this study. All participants, including HC, underwent a detailed neuropsychology assessment. It is reassuring that our electrophysiological measure (EC/EO bi-centroparietal synchronization) correlated with cognitive scores. Further work is, however, required to explore if this technique can detect cognitive impairment due to other causes.

This is the first time the ERR causality test has been used in the field of dementia. We have previously shown that this test has significant advantages over other measures of synchronization, such as coherence, due to its ability to detect linear as well as non-linear interactions [52]. In fact, coherence provides a normalized average level of synchronization estimated over a fixed period of time, which might reduce or even fail to reveal important dynamic changes and nonlinear interactions. The advantages associated with the use of non-linear analyses in this field are widely recognized [25,26,27], and very recently non-linear deficits on EEGs from patients with AD have been shown to involve the centroparietal areas [53]. Additionally, our method only requires short periods of EEG data.

The ERR-causality test procedure was carried out in both EO and EC states, in order to disentangle possible group-differences in the dynamic shift between resting-state conditions. This concept is not new and Pritchard and coworkers [26,28] have previously demonstrated something similar in patients with AD. In their work, these authors used a non-linear estimate, called D2, a reflection of the complexity of the cortical dynamics on EEG recordings, and revealed prominently increased complexity during the EO, compared to the EC state in healthy controls, but no significant differences between the two states in AD. The authors interpreted these data as a loss of brain responsivity to changing environmental stimuli. Work from the same group has also highlighted the positive impact on predictive classification of AD versus HC by implementing non-linear EEG measures [26].

This pilot study shows that using the ERR test, we can detect a large difference between AD patients and HC when computing the EO/EC ratio of bi-centroparietal synchronization. HC are able to desynchronize their EEGs by more than 50% during EO periods, whereas patients with AD can do so only by an average of 20%. Stam and colleagues [12] showed an analogous finding with reduced fluctuations on EEG synchronization in AD. These observations are of interest as there is evidence that a balanced and temporally precise pattern of synchronization and desynchronization is of paramount importance for normal cognitive function [15]. Our results suggest that estimates of EEG synchronization are state dependent (Figure 3) and it is important for EO and EC epochs to be analyzed separately.

EEG offers unique temporal resolution to assess functional brain network connectivity and, as cognitive brain function occurs within the millisecond range, it has unique characteristics for the development of a diagnostic tool for dementia [2,4]. The ERR-causality test has the advantage of being a parametric technique that measures accurately levels of synchronization between pairs of EEG electrodes, and can detect dynamic changes without making any assumptions about the stationarity of the signals [30]. This test does not require fitting of a complete model to produce its estimates and as a result it is fast and computationally not particularly demanding.

This pilot study has limitations. First, it was based on a small number of participants. However, a large number of clinical measures were put into practice to characterize both patient and control groups and to ensure that the AD patients were representative of the typical AD population of comparable severity. All participants, including the control sample, underwent detailed neuropsychology and brain imaging. Group analyses to assess cerebral atrophy and DMN connectivity revealed results consistent with the literature, demonstrating extensive parietal, temporal, mediotemporal/limbic GM decrease and loss of DMN connectivity in the posterior cingulate/retrosplenial cortex. Second, the groups were not well matched for education. This difference, however, does not represent a major issue, since it is well established that low education levels are a risk factor for the onset of AD, and, overall, it is normal to observe lower levels of education in AD patients than in healthy age-matched controls. Education was only controlled for through post-hoc analyses. Third, six patients were taking types of medication that can affect EEG signal; none of the patients were taking benzodiazepines that can cause an excess of fast activity [54]. The AChEI medications that some of our patients were receiving during the EEG recordings have been shown to reduce the amount of delta and theta activity, and increase the dominant alpha rhythm [55]. A case study has looked at memantine (three of our patients were on treatment with this medication) showing a reduction of pathological theta rhythms after several months [56]. Memantine is more frequently used in people with more severe AD and hence this patient group may have had more severe changes detectable on EEG. It is unlikely that any of the aforementioned medications could have influenced the findings of this study, since only 30% of our patient group was on this type of medication. However, further studies in the area of QEEG and response to anti-dementia medication are required.

Diagnostic criteria for AD include biological markers (such as cerebrospinal fluid and amyloid PET imaging), but these are invasive or expensive, and neither is widely available. We did not have these biomarkers available for this cohort, but have characterized them with structural and functional MRI and detailed neuropsychology and regular follow-up over at least three years. The QEEG ERR test used on resting state EEG recordings in this study is easy to use, non-invasive, economical to administer, and potentially portable. We argue that this novel QEEG test has the potential to become a useful diagnostic aid in the assessment of people with cognitive complaints [1]. It also has the potential to produce an electrophysiological AD biomarker. Devising a biomarker is a long and complicated process. Nonetheless, such a biomarker is urgently needed, both for the clinical management of AD, as well as for the selection of patients for clinical trials of new therapies.

The findings of this pilot work are encouraging as the ERR causality test shows a strong correlation between the EO/EC bi-centroparietal synchronization ratio and the neuropsychology measures of episodic memory, semantic processing, and visuo-constructive abilities. Alteration in our electrophysiological test does not correlate with age and hence it does not simply reflect age-related brain changes. Previous studies using QEEG have shown good correlation with MMSE [57]. However, these studies did not have detailed neuropsychology results on healthy volunteers, which is a strength of our study.

Our major future objective is to assess the value of this test in the prodromal phase of the disease (mild cognitive impairment due to AD, or presymptomatic AD according to Dubois proposed criteria [58]). This may be challenging, as, at this stage, changes in synchronization and connectivity are reported to be different than those observed in AD [13,59,60,61], most likely as a consequence of either compensatory or maladaptive mechanisms [62]. Further studies will require longitudinal data collection with detailed neuropsychology, clinical and radiological follow-ups. This study was designed to assess if our QEEG method could distinguish a well-characterized group of participants with dementia due to Alzheimer’s disease from healthy controls.

In summary, these preliminary findings indicate that the EEG-based ERR-causality test is capable of separating patients diagnosed with a dementia due to Alzheimer’s disease from HC. Particularly, the dynamic shift between EO and EC states seems to be lost in AD in the parietal areas. It is also of interest that the parietal areas showing dysfunction or loss of connectivity in the DMN on fMRI also show severe abnormalities on our EEG measure. Synchronization during EO state, measured with the ERR test, is increased in bi-centroparietal regions in people with AD. Neuronal synchronization between brain structures constitutes a flexible mechanism to coordinate information flow in the cerebral cortex across several spatiotemporal scales and may be essential for cognition [63]. Our findings reaffirm the concept that a balanced and temporally precise pattern of synchronization and desynchronization is pertinent to cognitive function [15]. We show that patients with AD have very little differences in synchronization between EO versus EC biological states, when compared to the results obtained from healthy controls. Furthermore, this ratio strongly correlates with neuropsychological tests of memory and praxis. In patients with AD, brain networks appear to be locked at a relatively fixed level of synchronization without the dynamic variations and responsiveness to external stimuli found in healthy controls. This finding could potentially reflect the neurophysiological correlate of defective default mode activity modulation in AD. Although causality between these two findings cannot be inferred, it is reasonable to postulate that the two methods capture different facets of the same underlying cerebral network dysfunction.

## Figures and Tables

**Table 1:** Demographic data, Structural MRI & Neuropsychology results.

Variable	AD Patients	Healthy Controls	*p*
*Demographics*			
Age at MRI scan (years)	63.95 (8.34)	67.35 (11.79)	0.429
Education (years)	11.75 (2.05)	15.20 (2.98)	<0.001
Gender (f/m)	10/10	11/9	0.752
Mini Mental State Examination (max. 30)	20.10 (4.00)	28.20 (1.36)	<0.001
*Brain Structure-Absolute Volumes*			
Left Hippocampus (mL)	2.08 (0.48)	2.59 (0.24)	<0.001
Right Hippocampus (mL)	2.21 (0.48)	2.61 (0.34)	0.007
Grey Matter (mL)	550.24 (83.00)	627.19 (61.11)	0.003
White Matter (mL)	396.76 (58.17)	418.40 (39.01)	0.192
*Brain Structure-Fractional Volumes*			
Grey Matter	0.38 (0.05)	0.43 (0.05)	0.002
White Matter	0.28 (0.04)	0.29 (0.02)	0.121
*Cognition*			
Letter Fluency Test *	20.79 (13.77)	48.40 (14.21)	<0.001
Category Fluency Test *	22.00 (9.90)	56.80 (13.56)	<0.001
Prose Memory Test-Immediate Recall (max. 24) *	4.37 (3.62)	15.15 (3.53)	<0.001
Prose Memory Test-Delayed Recall (max. 24) *	4.42 (4.31)	19.65 (2.28)	<0.001
Stroop Test-Time Interference *	29.61 (21.09)	13.70 (4.20)	0.016
Stroop Test-Error Interference *	12.22 (10.59)	0.00 (0.00)	<0.001
Visuoconstructional Praxis Test (max. 14)	7.55 (3.94)	12.35 (1.53)	<0.001

Means and standard deviations are indicated. Mann-Whitney *U* test between groups were run for each comparison. A Chi-Square test was used for gender. One AD patient did not complete the tests marked by an asterisk.

**Table 2:** QEEG results-ERR-causality test.

EEG Index	AD Median (IQR)	FTD Median (IQR)	HC Median (IQR)	AD vs. HC	AD vs. FTD	HC vs. FTD
Bi-centroparietal (C3–P3 to C4–P4)						
Eyes closed 95% CI top level	0.53 (0.071)	0.14 (0.27)	0.485 (0.53)	*p* = 0.968	*p* = 0.037	*p* = 0.018
Eyes open 95% CI top level	0.435 (0.41)	0.075 (0.03)	0.15 (0.17)	*p* < 0.0001	*p* < 0.0001	*p* = 0.018
EO/EC ratio	0.784 (0.386)	0.586 (0.386)	0.371 (0.346)	*p* < 0.0001	*p* = 0.241	*p* = 0.575
Bi frontal (F3–F7 to F4–F8)						
Eyes closed 95% CI top level	0.185 (0.27)	0.07 (0.04)	0.11 (0.11)	*p* = 0.149	*p* = 0.018	*p* = 0.081
Eyes open 95% CI top level	0.09 (0.1)	0.08 (0.04)	0.09 (0.08)	*p* = 0.64	*p* = 0.431	*p* = 0.431

EO: Eyes-open; EC: eyes-closed; CI: Confidence interval; IQR: Interquartile range (distance between the 5th percentile and the 95th percentile). Median and interquartile ranges are shown, as the data was not normally distributed. EO/EC Bi-frontal synchronization not shown.

## Appendix A. The Error Reduction Ratio (ERR) Causality tEst

This is a test that we have previously introduced which overcomes most of the disadvantages of existing methods [30,53,64]. The aim of this approach is to characterize the causal interaction over time between two signals, denoted by X and Y. At a specific time the following possibilities can occur, signal X causes Y, Y causes X, X and Y are coincident, there is no interaction, or bidirectional interactions occur between them. Both X and Y may have been caused by an input u but here u is unknown and un-measurable. For a complex system, the causality is often time varying and the interactions are often dynamic and nonlinear.

Initially, construct a candidate term set which is typically constructed by past information of Y, for example y(t − 1), y(t − 2), ... and past information of X, for example x(t − 1), x(t − 2), ... with a specific model order d. Apply the adaptive-forward-OLS algorithm which has been derived from the orthogonal least squares (OLS) algorithm [44] and compute ERR and the penalized error-to-signal ratio PESR) [45] value for each candidate term. ERR indicates how much of the variance change in the system response is caused by the considered term, in a percentage form. If the significant terms selected by this algorithm based on values of ERR include any term from the past information of X, this indicates that signal X leads Y during the considered time duration (t − h/2, t + h/2) where h denotes the sampling window size - this was selected at 1000 data points (corresponding to 0.5s) for this work. The ERR-causality from X to Y at time t, expressed as F_x_ > y(t) is then defined as 1. If no component from the past information of X is included in the selected significant terms, this indicates that X has no interaction with Y during [t − h/2, t + h/2] and F_x_ > y(t) is defined to be 0. The strength of F_x_ > y(t) can be estimated using the summed ERR values of all the selected terms from past information of X, the maximum strength being 1. The time shift of X > Y is defined as the time lag of X in the first term ranked by the values of ERR. The key advantage of this test is that it can be applied to both linear and nonlinear dynamic systems. Unlike the well-established Granger-based tests (Granger, 1969) to quantify the causality between two signals, the ERR-Causality method does not depend on the full knowledge or the estimation of a complete and unbiased system model. By exploiting an important property of the ERR-Causality test, part of the orthogonal least squares algorithm, it is shown that the causal flow can be detected even when the model is incomplete. This is a significant advantage when the underlying system is nonlinear and dynamic and the measurements may be noisy, because a complete and full model including a nonlinear noise model, which would normally be required to yield unbiased model estimates, is not required and indeed not even the full parameter estimates are used in the test. These advantages mean that the test is relatively easy to apply and can be used to track fast transitions between causal effects to detect the direction of linear or nonlinear causal interactions, determine the strength of these interactions, and provide an estimate of the time lag shift between two EEG signals in this instance. Furthermore, linear and nonlinear interactions can be separated and analysed independently with this method as there is evidence that the nonlinear dimension appears to be important in the pathophysiology of AD and cannot be assessed by conventional qEEG methods [2,25].
